# Magnetized suspended carbon nanotubes based nanofluid flow with bio-convection and entropy generation past a vertical cone

**DOI:** 10.1038/s41598-019-48645-9

**Published:** 2019-08-21

**Authors:** Muhammad Ramzan, Mutaz Mohammad, Fares Howari

**Affiliations:** 1 Department of Computer Science, Bahria University, 44000 Islamabad, Pakistan; 2 Department of Mechanical Engineering, Sejong University, Seoul, 143-747 Korea; 3 Department of Mathematics & Statistics, College of Natural and Health Sciences, Zayed University, 144543 Abu Dhabi, UAE; 4 College of Natural and Health Sciences, Zayed University, 144543 Abu Dhabi, UAE

**Keywords:** Mechanical engineering, Applied mathematics

## Abstract

The captivating attributes of carbon nanotubes (CNT) comprising chemical and mechanical steadiness, outstanding electrical and thermal conductivities, featherweight, and physiochemical consistency make them coveted materials in the manufacturing of electrochemical devices. Keeping in view such exciting features of carbon nanotubes, our objective in the present study is to examine the flow of aqueous based nanofluid comprising single and multi-wall carbon nanotubes (CNTs) past a vertical cone encapsulated in a permeable medium with convective heat and solutal stratification. The impacts of heat generation/absorption, gyrotactic-microorganism, thermal radiation, and Joule heating with chemical reaction are added features towards the novelty of the erected model. The coupled differential equations are attained from the partial differential equations by exercising the local similarity transformation technique. The set of conservation equations supported by the associated boundary conditions are worked out numerically by employing bvp4c MATLAB function. The sway of numerous appearing parameters in the analysis on the allied distributions is scrutinized and the fallouts are portrayed graphically. The physical quantities of interest including Skin friction coefficient, the rate of heat and mass transfers are assessed versus essential parameters and their outcomes are demonstrated in tabulated form. It is witnessed that the velocity of the fluid decreases for boosting values of the magnetic and suction parameters in case of both nanotubes. Moreover, the density of motile microorganism is decreased versus larger estimates of bio-convection constant. A notable highlight of the presented model is the endorsement of the results by matching them to an already published material in the literature. A venerable harmony in this regard is achieved.

## Introduction

The topic of nanofluid flow has earned exceptional consideration in the last two decades due to its standing in copious industrial and engineering applications. The scientists and researchers have not only explored the surprising thermal characteristics of nanofluids but also suggested the reasons for the enhancement of thermal conductivity of nanofluids. Because of surprising features of nanofluids, these are being considered as future coolants for computers and obviously the reliable coolants for nuclear reactors. An amalgamation of nanofluids and biotechnological mechanisms may offer promising applications in biological sensors, pharmaceuticals and agricultural. In the field of biotechnology, numerous nanomaterials like nanofibers, nanoparticles, nanostructures, and nanowires are in practice. The Nano biotechnological has a potential market and one can foresee that the future of such products is very bright. Likewise, in the area of biomedical devices and procedures, the importance of nano and micro-fluidics can’t be denied. The magnetic nanofluids own both magnetic and liquid characteristics and have applications in varied fields like optical switches, tunable optical fiber filters, modulators, and optical gratings. The magnetic nanoparticles have an imperative role in medicine, cancer therapy, loudspeakers, and sink-float separation. The concept of renewable energy generation is the core subject of today’s era across the world. Solar energy is the prime source of renewable energy with minimal ecological pollution. One can obtain energy, electricity, and water from the solar source directly. Researchers are of the opinion that solar collection processes can be triggered by the insertion of nanoparticles in the fluids. In several industrial processes, such as power transportation and manufacturing, cooling and heating of fluids is the prime requirement. Improvement in the cooling process in high energy devices is the need of the day. But ordinary heat transfer liquids like water, engine oil, and ethylene glycol possess poor heat transfer characteristics and do not meet the cooling requirements of the industry. Instead, if we talk about the thermal conductivities of the metals which are relatively much higher than those of conventional aforementioned fluids. Thus, a combination of both ingredients to form an excellent heat transfer medium that performs as fluid can be comprehended. Choi^1^ ground-breaking idea of nanofluid by addition of metallic nanoparticles (with size less than 100 nm in diameter) into the conventional fluids like water for the enhancement of the thermal conductivity has revolutionized the engineering and industrial world. Then Buongiorno^2^ deliberated the characteristics of nanofluid keeping in view the Brownian motion and thermophoresis. The flow of nanofluid with H2O as base fluid over a convectively heated surface is studied by Makinde and Aziz^3^. The nanofluid flow in an enclosure with impacts of non-isothermal temperature distribution and natural convection was studied by Oztop *et al*.^4^. Turkyilmazoglu^5^ found an exact analytical solution of hydro-magnetic viscous fluid flow past a spongy surface with thermal slip condition. The flow of nanofluids, with water base fluid in a lid-driven cavity with crimped surfaces in attendance of mixed convection, was studied by Cho *et al*.^6^. Sheikholeslami and Ganji^7^ discussed the analytical solution of copper-water nanofluid squeezing flow amid two parallel plates using Homotopy perturbation method. The flow of non-Newtonian fluid flow with the amalgamation of Sodium alginate and Titanium oxide past two coaxial cylinders in a spongy medium was discussed by Hatami and Ganji^8^. The flow of time-dependent nanofluid by a vertical surface in the attendance of mixed convection and thermal radiation was discussed by Turkyilmazoglu and Pop^9^. Sheikholeslami *et al*.^10^ deliberated analytical solution of nanofluid flow past a spongy channel with effects of magnetohydrodynamics. Ibrahim and Makinde^11^ inspected the flow of nanofluid by a vertical plate influenced by thermal and solutal stratification. Turkyilmazoglu^12^ examined the flows of some nanofluids with numerous nanoparticles like copper, silver, copper oxide, titanium oxide and alumina with water as base fluid with two types of temperature boundary conditions. The flow of nanofluid past a penetrable rotating disk in attendance of the magnetohydrodynamics with the impact of entropy generation was conducted by Rashidi *et al*.^13^. Lin *et al*.^14^ deliberated the flow of pseudo-plastic nanofluid with Marangoni convection flow, variable thermal conductivity and thermal radiation. Hussain *et al*.^15^ described the series solutions of the flow of the Casson nanofluid flow with convective heat and mass boundary conditions over an exponentially stretching surface. The flow of 3D viscoelastic nanofluid under the influences of chemical reaction and magnetohydrodynamics was analyzed by Ramzan and Bilal^16^. Lu *et al*.^17^ debated numerically the flow of three-dimensional nanofluid with the binary chemical reaction, gyrotactic microorganism, activation energy and anisotropic slip along with a moving plate. The flow of viscous nanofluid with impacts of motile gyrotactic micro-organisms and nonlinear thermal radiation is deliberated by Ramzan *et al*.^18^. Additional effects of chemical reaction with Joule heating and slip boundary condition are also considered. Fewer recent investigations highlighting nanofluids may be found in^19–25^.

The movement of an electrically charged fluid owing to a magnetic field is characterized as magnetohydrodynamics (MHD). The idea of MHD got the attention in 1918 as stated by Rossow^26^, after the coined work of an electromagnetic pump that leads to the definition of Hartmann number. Alfven^27^ described that an electric current will be produced by an electromotive force (e.m.f.) which is generated by the fluid that is under the influence of a constant magnetic field. Because of this e.m.f., a kind of electric current is induced while the other generates the Lorentz force. Because of the magnetic field, these electric currents produce mechanical forces that alter the fluid’s state of motion and eventually electromagnetic-hydrodynamic wave is produced. Since that time, scientists and researchers have worked on many applications of MHD like MHD generators, metallurgy, MHD pumps’ designs and dispersion of metals, etc. Geophysics does possess the physiognomies of MHD owing to the association of magnetic field and conducting fluid. In the area of planetary and stellar, the problem of MHD convection has vital significance. The flow of Micropolar nanofluid under the influence of MHD, Buoyancy forces, and activation energy with binary chemical reaction accompanied by double stratification is examined by Ramzan *et al*.^28^. Lu *et al*.^29^ analyzed the flow of MHD 3D Oldroyd-B fluid associated with the impacts of nonlinear thermal radiation, variable thermal conductivity, and homogeneous-heterogeneous reactions analytically. The flow of Jeffrey MHD nanofluid flow with nonlinear radiative heat flux is studied by Ijaz *et al*.^30^. Recently, Zhang *et al*.^31^ found numerical solution Fractional Oldroyd-B nanofluid between two concentric cylinders using Finite difference technique amalgamated with L1-algorithm and many therein^32–35^.

Carbon nanotubes (CNTs) are cylindrical shaped tubes with irreplaceable characteristics like good thermal conductivity, and tremendous potency makes them highly desirable materials in varied applications like microwave amplifier, optics, drug delivery, nanotubes transistors, and prostheses and many other areas^36–39^. CNTs are available in the form of single-wall carbon nanotubes (SWCNTs) and multi-wall carbon nanotubes (MWCNTs). Iijima introduced MWCNTs in 1991. Then in 1993, Donald Bethune gave the idea of SWCNTs. Ramasubramaniam *et al*.^40^ in his exploration examined that SWCNTs are quite helpful in electrical conductivity applications. Xue^41^ identifies that composite nanotubes are essential to improve thermal conductivity. The flow of peristaltic nanofluid comprising of SWCNTs and blood is discussed by Nadeem *et al*.^42^. The influence of h-h reactions in a 3D flow of CNTs is deliberated by Hayat *et al*.^43^. Shah *et al*.^44^ inspected the rotating flow of carbon nanotubes under the influence of heat generation/absorption and nonlinear thermal radiation past a linearly stretching surface. Recent studies highlighting nanofluids impacts in various scenarios may be found in^33,45–54^.

The literature quoted above reveals that numerous articles are available pertaining to nanofluids with varied geometries but less literature exists relating to nanofluid flows with CNTs over a cone. Further, this exploration becomes unique when above-mentioned characteristics are supported with Joule heating, entropy generation, chemical reaction, Heat generation/absorption, and solutal stratification boundary condition. The solution of the erected model is obtained numerically via bvp4c MATLAB built-in function.

## Mathematical Modeling

**Figure 1:**
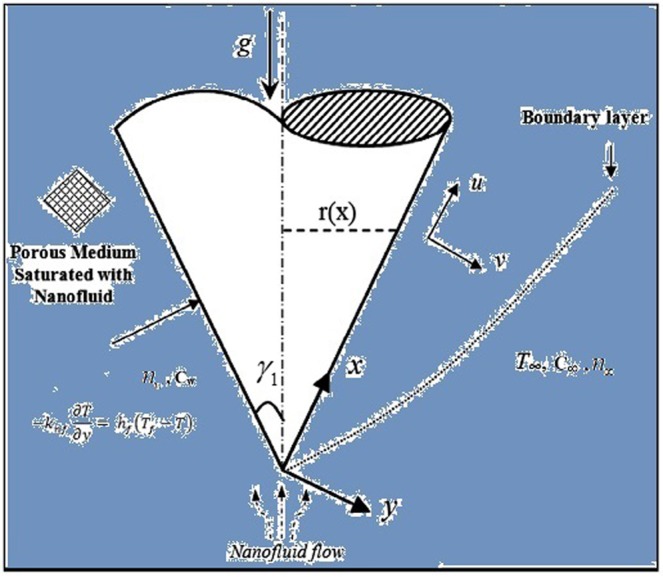
Schematic model representing the flow problem.

We assume a water based nanofluid flow with CNTs over a vertical cone in a permeable medium. The additional effects accompanied the model are Heat generation/absorption, solutal stratification, Joule heating, chemical reaction with entropy generation. The flow is induced along the *x*–axis and a magnetic field is applied along the *y*–axis as shown in Fig. 1. The equations resulting from the above assumptions are modeled as^55^:

1 $$\frac{\partial (ru)}{\partial x}+\frac{\partial (rv)}{\partial y}=0,$$

2 $$\begin{array}{rcl}u\frac{\partial u}{\partial x}+v\frac{\partial u}{\partial y} & = & \frac{{\mu }_{nf}}{{\rho }_{nf}}\frac{{\partial }^{2}u}{\partial {y}^{2}}-\frac{{\mu }_{nf}}{{\rho }_{nf}}\frac{1}{K}u+g[\beta (T-{T}_{\infty })\\  &  & -\,{\beta }^{\ast }(C-{C}_{\infty })-{\beta }^{\ast }\gamma (n-{n}_{\infty }){\rm{\Delta }}\rho ]\,\cos \,{\gamma }_{1}\\  &  & -\,\frac{\sigma {{B}_{0}}^{2}}{{\rho }_{nf}}u,\end{array}$$

3 $$u\frac{\partial T}{\partial x}+v\frac{\partial T}{\partial y}={\alpha }_{nf}\frac{{\partial }^{2}T}{\partial {y}^{2}}-\frac{1}{{(\rho {c}_{p})}_{nf}}\frac{\partial {q}_{r}}{\partial y}+\frac{{Q}_{0}}{{(\rho {c}_{p})}_{nf}}(T-{T}_{\infty })+\frac{\sigma {{B}_{0}}^{2}}{{(\rho {c}_{p})}_{nf}}{u}^{2}$$

4 $$u\frac{\partial C}{\partial x}+v\frac{\partial C}{\partial y}={D}_{m}\frac{{\partial }^{2}C}{\partial {y}^{2}}-{K}_{r}(C-{C}_{\infty }),$$

5 $$u\frac{\partial n}{\partial x}+v\frac{\partial n}{\partial y}+\frac{b{W}_{c}}{{C}_{w}-{C}_{0}}\frac{\partial }{\partial y}(n\frac{\partial C}{\partial y})={D}_{n}\frac{{\partial }^{2}n}{\partial {y}^{2}},$$

with the corresponding boundary conditions

6 $$\begin{array}{l}v={V}_{1},u=0,-\,{k}_{nf}\frac{\partial T}{\partial y}={h}_{f}({T}_{f}-T),C={C}_{w}={C}_{0}+dx,n={n}_{w},\,{\rm{at}}\,y=0,\\ u\to 0,T\to {T}_{\infty },C\to {C}_{\infty }={C}_{0}+ex,n\to {n}_{\infty },\,{\rm{as}}\,y\to \infty .\end{array}$$

Here, (*μ*_*nf*_, *μ*_*f*_), (${\rho }_{CNT}$, ${\rho }_{f}$), (*β*, β*), *γ*_1_, *B*_0_, *α*_*nf*_, *q*_*r*_, *Q*_0_, ${(\rho {c}_{p})}_{nf}$, and ${(\rho {c}_{p})}_{f}$, *D*_*m*_, *K*_*r*_, *W*_*c*_, *D*_*n*_, (*k*_*f*_, *k*_*nf*_, *k*), *h*_*f*_, (*d*, *e*), and *V*_0_ represent dynamic viscosity, density, thermal and solutal expansion coefficients, cone half-angle, magnetic field of strength, modified thermal diffusivity, thermal radiation coefficient, Dimensional heat generation/absorption parameter, heat capacities, Brownian diffusion coefficient, rate of chemical reaction, maximum cell swimming speed, diffusivity of microorganisms, thermal conductivity, convective parameter, reference temperature and concentration dimensionless constants and suction/injection parameter respectively.

Table 1 is erected to depict the characteristics of water and CNTs of both types.

**Table 1:** Characteristics of H_2_O and both types of CNTs *i*.*e*., SWCNTs, and MWCNTs.

	C_p_ (J/kg K)	*ρ* (kg/m^3^)	*k* (W/mK)
H_2_O	4179	997	0.613
SWCNTs	425	2600	6600
MWCNTs	796	1600	3000

Thermal conductivity and effective density of the nanofluid are given by:

7 $$\begin{array}{c}{\mu }_{nf}=\tfrac{{\mu }_{f}}{{(1-\varphi )}^{2.5}},\,{v}_{nf}=\tfrac{{\mu }_{nf}}{{\rho }_{nf}},\\ {\rho }_{nf}=(1-\varphi ){\rho }_{f}+\varphi {\rho }_{CNT},\,{\alpha }_{nf}=\tfrac{{k}_{nf}}{{\rho }_{nf}{({c}_{p})}_{nf}},\\ \frac{{k}_{nf}}{{k}_{f}}=\frac{(1-\varphi )+2{\varphi }\tfrac{{k}_{CNT}}{{k}_{CNT}-{k}_{f}}\,\mathrm{ln}(\tfrac{{k}_{CNT}+{k}_{f}}{2{k}_{f}})}{(1-\varphi )+2{\varphi }\tfrac{{k}_{f}}{{k}_{CNT}-{k}_{f}}\,\mathrm{ln}(\tfrac{{k}_{CNT}+{k}_{f}}{2{k}_{f}})}.\end{array}$$

The similarity transformations are defined as

8 $$\begin{array}{rcl}\eta  & = & \frac{y}{x}R{a}_{x}^{1/4},{\rm{\Psi }}=\alpha R{a}_{x}^{1/4}f(\eta ),\,\theta (\eta )=\frac{T-{T}_{\infty }}{{T}_{w}-{T}_{\infty }},\\ g(\eta ) & = & \frac{C-{C}_{\infty }}{{C}_{w}-{C}_{0}},h(\eta )=\frac{n-{n}_{\infty }}{{n}_{w}-{n}_{\infty }},\end{array}$$

Equation (1) is satisfied and equations (3) to (8) take the form

9 $$\begin{array}{l}f\prime\prime\prime +\frac{1}{\Pr }{(1-{\varphi })}^{2.50}(1-{\varphi }+{\varphi }\tfrac{{\rho }_{CNT}}{{\rho }_{f}})\{3ff^{\prime\prime} -\frac{1}{2}f{^{\prime} }^{2}\}\\ \,-\,{k}_{1}f^{\prime} -{(1-{\varphi })}^{2.5}Mf^{\prime} +\,{(1-{\varphi })}^{2.50}(1-{\varphi }+{\varphi }\tfrac{{\rho }_{CNT}}{{\rho }_{f}})[\theta -{N}_{r}g-{R}_{b}h]=0,\end{array}$$

10 $$\frac{{k}_{nf}}{{k}_{f}}(1+{R}_{d})\theta ^{\prime\prime} +\frac{3}{4}[1-{\varphi }+{\varphi }\tfrac{{(\rho {C}_{p})}_{CNT}}{{(\rho {C}_{p})}_{f}}]f\theta ^{\prime} +\gamma \theta +{\rm{\Pr }}\,EcMf{^{\prime} }^{2}=0,$$

11 $$g^{\prime\prime} +\frac{3}{4}{S}_{c}fg^{\prime} -{S}_{c}nf^{\prime} -{C}_{r}g=0,$$

12 $$h^{\prime\prime} +\frac{3}{4}{L}_{b}fh^{\prime} -{P}_{e}(h^{\prime} g^{\prime} +(h+\delta )g^{\prime\prime} )=0,$$

13 $$\begin{array}{l}f(0)={V}_{0},f^{\prime} (0)=0,\frac{{k}_{nf}}{{k}_{f}}\theta ^{\prime} (0)=-\,{B}_{1}(1-\theta (0)),g(0)=1-n,h(0)=1,\\ f^{\prime} (\infty )\to 0,\theta (\infty )\to 0,g(\infty )\to 0,h(\infty )\to 0.\end{array}$$

The parameters in non-dimensional form are stated as:

14 $$\begin{array}{c}\Pr =\tfrac{{\upsilon }_{f}}{\alpha },{k}_{1}=\tfrac{{x}^{2}}{KR{a}_{x}^{1/2}},M=\tfrac{\sigma {B}_{0}^{2}{x}^{2}}{{\mu }_{f}R{a}_{x}^{1/2}},{S}_{c}=\tfrac{\alpha }{{D}_{m}},n=\tfrac{e}{d},\gamma =\tfrac{{Q}_{o}{x}^{2}}{(\rho {c}_{p})R{a}_{x}^{1/2}},\\ {L}_{b}=\tfrac{\alpha }{{D}_{n}},{R}_{d}=\tfrac{16{T}_{\infty }^{3}{\sigma }^{\ast }}{3{k}^{\ast }{k}_{nf}},{N}_{r}=\tfrac{{\beta }^{\ast }({C}_{w}-{C}_{0})}{\beta ({T}_{f}-{T}_{\infty })},{R}_{b}=\tfrac{{\beta }^{\ast }\gamma {\rm{\Delta }}\rho {\rm{\Delta }}{n}_{w}}{\beta ({T}_{f}-{T}_{\infty })},\\ {C}_{r}=\tfrac{{K}_{r}{x}^{2}}{{D}_{m}R{a}_{x}^{1/2}},{B}_{1}=\tfrac{{h}_{f}x}{R{a}_{x}^{1/4}{k}_{f}},{P}_{e}=\tfrac{b{W}_{c}}{{D}_{n}},\delta =\tfrac{{n}_{\infty }}{{n}_{w}-{n}_{\infty }}.\end{array}$$

Here, Pr, *k*_1_, *M*, *N*_*r*_, *R*_*b*_, *R*_*d*_, *E*_*c*_, *γ*, *S*_*c*_, *C*_*r*_, *L*_*b*_, *P*_*e*_, *δ*, *B*_1_, and *n* characterize Prandtl number, Porous parameter, magnetic parameter, buoyancy ratio parameter, Bio-convection Rayleigh number, Radiation parameter, heat generation/absorption parameter, Schmidt number, Chemical reaction parameter, bio-convection Lewis number, Bio-convection constant, Boit number and solutal stratification respectively.

The physical quantities like drag coefficient, local Nusselt and Sherwood numbers, and local density of motile microorganisms are given by:

15 $${C}_{f}=\tfrac{{\tau }_{w}}{\rho {U}_{\infty }^{2}},N{u}_{x}=\tfrac{x{q}_{w}}{{k}_{f}({T}_{w}-{T}_{\infty })},S{h}_{x}=\tfrac{x{q}_{m}}{{D}_{m}({C}_{w}-{C}_{0})},N{n}_{x}=\tfrac{x{q}_{n}}{{D}_{n}({n}_{w}-{n}_{\infty })},$$

In dimensionless form, the above physical quantities are evaluated as:

16 $$\begin{array}{rcl}{C}_{f}R{a}_{x}^{1/4} & = & \tfrac{1}{{(1-\varphi )}^{2.5}}f^{\prime\prime} (0),\\ N{u}_{x}R{a}_{x}^{-1/4} & = & -\,\frac{{k}_{nf}}{{k}_{f}}(1+{R}_{d})\theta ^{\prime} (0),\\ S{h}_{x}R{a}_{x}^{-1/4} & = & -\,g^{\prime} (0),\\ N{n}_{x}R{a}_{x}^{-1/4} & = & -\,h^{\prime} (0).\end{array}$$

Table 2 shows the resemblance of the present results with Khan *et al*.^56^ for numerous estimates of $\varphi$ in limiting case. An exception concurrence between both results is found.

**Table 2:** Comparison with Khan *et al*.^56^ in limiting case for the values of $\varphi$ versus $f^{\prime\prime} (0)$ and $-\,\theta ^{\prime} (0)$.

*ϕ*	*f*″(0)				−*θ*′(0)			
	Khan *et al*.^56^		Existing Results		Khan *et al*.^56^		Existing Results	
	SWCNT	MWCNT	SWCNT	MWCNT	SWCNT	MWCNT	SWCNT	MWCNT
0.01	0.33894	0.33727	0.338996	0.337275	1.10553	1.07905	1.105529	1.079048
0.1	0.40811	0.39008	0.408111	0.390076	4.80627	4.27718	4.806269	4.277177
0.2	0.50452	0.46466	0.504521	0.464661	12.30317	10.56783	12.30316	10.56780

## Entropy Generation

The model representing the entropy generation is given by:

17 $$\begin{array}{rcl}{S\prime\prime\prime }_{gen} & = & \mathop{\underbrace{\frac{{k}_{nf}}{{{T}_{\infty }}^{2}}[1+\frac{16{{T}_{\infty }}^{3}{\sigma }^{\ast }}{3{k}^{\ast }{k}_{nf}}]{(\frac{\partial T}{\partial y})}^{2}}}\limits_{I}\\  &  & +\,\mathop{\underbrace{\frac{{\mu }_{nf}}{{T}_{\infty }}{(\frac{\partial u}{\partial y})}^{2}+\frac{\sigma }{{T}_{\infty }}{{B}_{0}}^{2}{u}^{2}+\frac{{\mu }_{nf}}{{T}_{\infty }K}{u}^{2}}}\limits_{II}\\  &  & +\,\mathop{\underbrace{\frac{RD}{{C}_{\infty }}{(\frac{\partial C}{\partial y})}^{2}+\frac{RD}{{T}_{\infty }}(\frac{\partial T}{\partial y})(\frac{\partial C}{\partial y})}}\limits_{III},\end{array}$$

In Eq. (17), entropy is consisting of three terms viz. *i)* I (heat transfer irreversibility) *ii)* II (fluid friction irreversibility) and *iii)* III (diffusion irreversibility). The entropy generation *N*_*G*_ is given by:

18 $${N}_{G}=\frac{{S\prime\prime\prime }_{gen}}{{S\prime\prime\prime }_{0}}.$$

Here, ${S\prime\prime\prime }_{gen}$ is entropy generation rate and ${S\prime\prime\prime }_{0}$ the characteristic entropy generation rate represented by:

19 $$\begin{array}{rcl}{N}_{G} & = & \frac{{k}_{nf}}{{k}_{f}}(1+R)R{a}_{x}\theta {^{\prime} }^{2}\\  &  & +\,\frac{1}{{(1-\varphi )}^{2.5}}\frac{BrR{a}_{x}}{\alpha }(f{^{\prime\prime} }^{2}+{k}_{1}f{^{\prime} }^{2})+\frac{R{a}_{x}BrM}{\alpha }f{^{\prime} }^{2}\\  &  & +\,\lambda {(\frac{\zeta }{\alpha })}^{2}R{a}_{x}g{^{\prime} }^{2}+\frac{\zeta }{\alpha }R{a}_{x}\lambda \theta ^{\prime} g^{\prime} ,\end{array}$$

where

20 $$\alpha =\frac{{\rm{\Delta }}T}{{T}_{\infty }},Br=\frac{{\mu }_{f}{u}_{w}}{{k}_{f}{\rm{\Delta }}T},\zeta =\frac{{\rm{\Delta }}C}{{C}_{\infty }},\lambda =\frac{RD{C}_{\infty }}{{k}_{f}}.$$

Here, *Ra*_*x*_, *Br*, *λ*, *ζ*, and *α* represent Reynold number, Brinkman number, diffusive constant parameter, concentration difference parameter, and temperature difference parameter respectively.

## Results and Discussion

**Figure 2:**
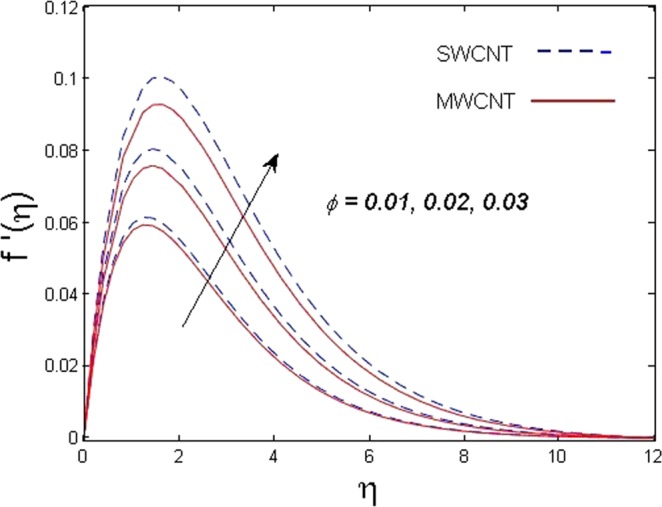
Outcome of $\varphi$ on $f^{\prime} (\eta )$.

**Figure 3:**
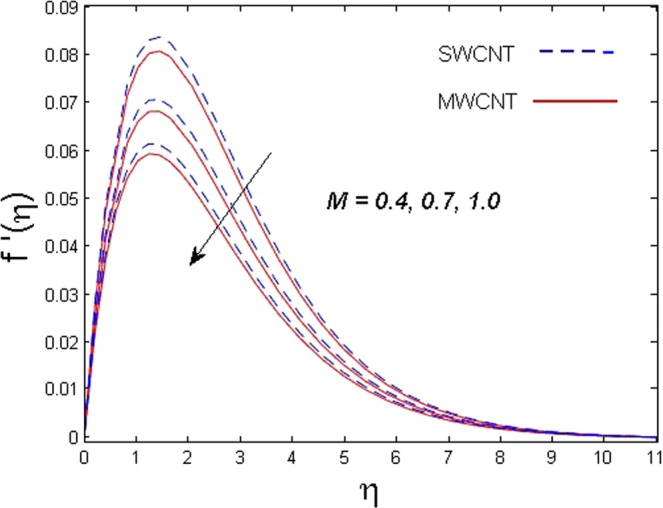
Outcome of *M* on $f^{\prime} (\eta )$.

**Figure 4:**
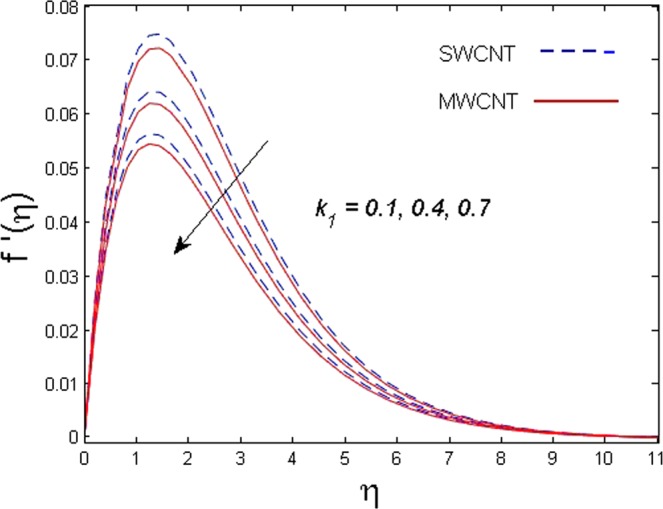
Outcome of *k*_1_ on $f^{\prime} (\eta )$.

**Figure 5:**
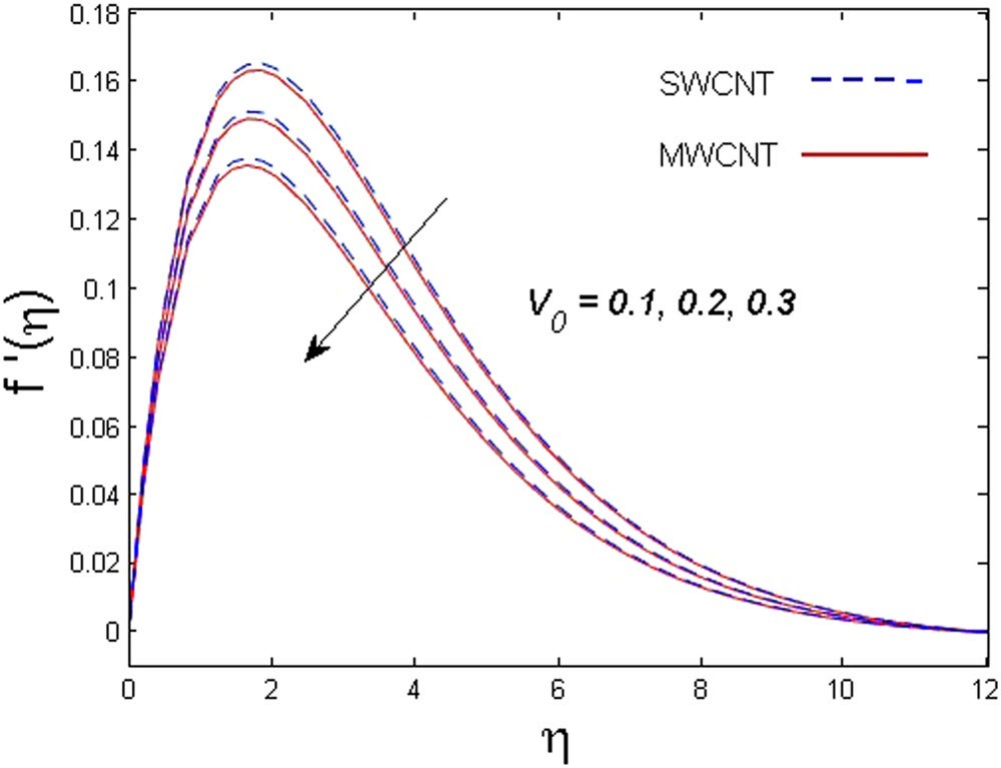
Outcome of v_0_ on $f^{\prime} (\eta )$.

**Figure 6:**
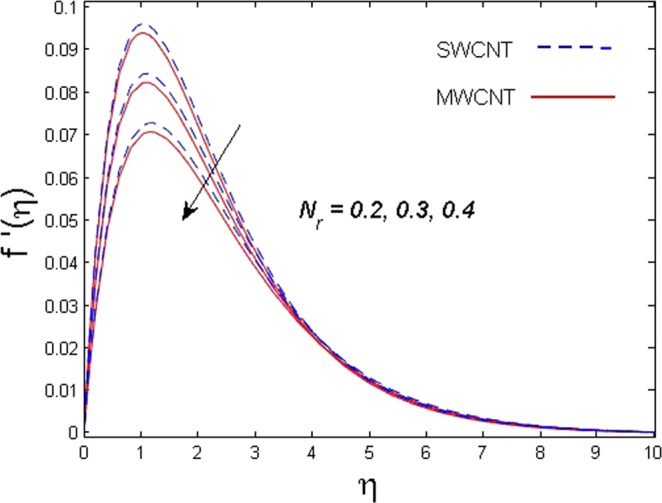
Outcome of *N*_*r*_ on $f^{\prime} (\eta )$.

**Figure 7:**
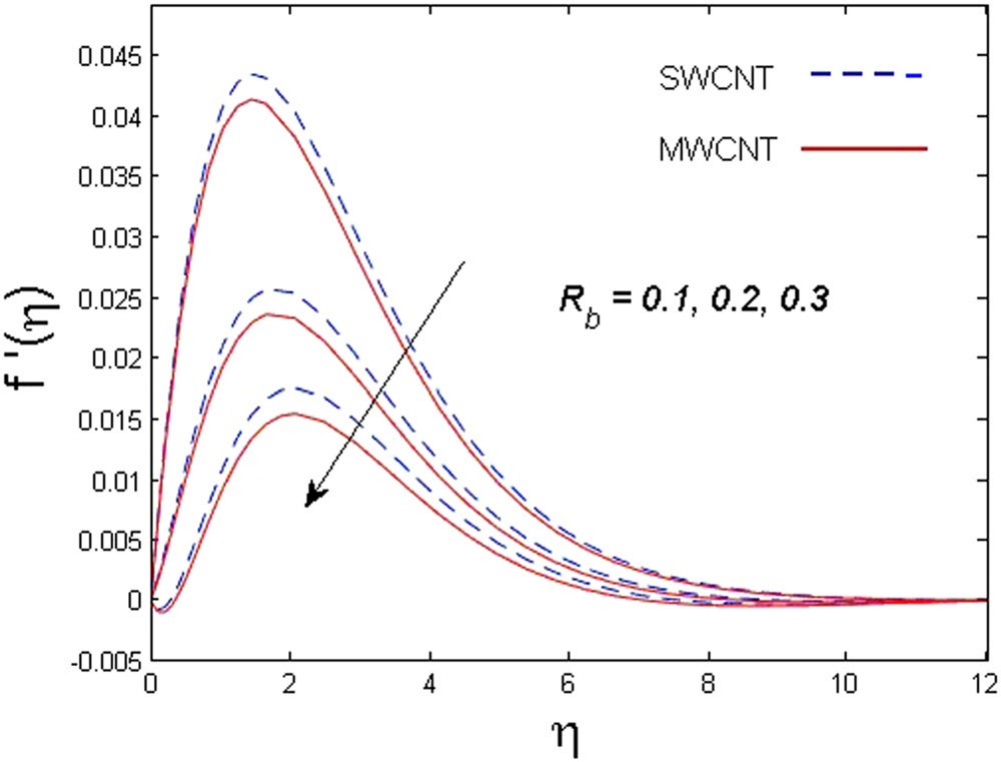
Outcome of *R*_*b*_ on $f^{\prime} (\eta )$.

**Figure 8:**
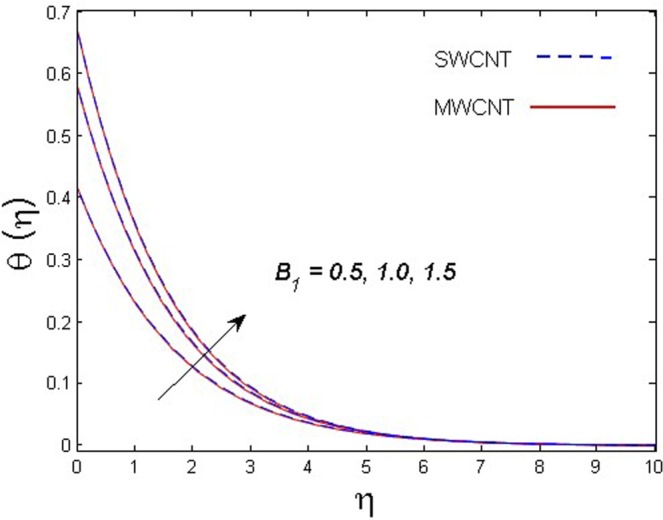
Outcome of *B*_1_ on $\theta (\eta )$.

**Figure 9:**
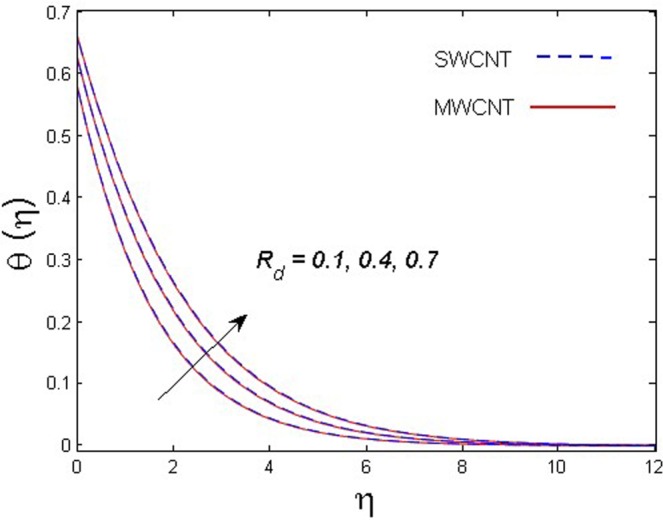
Outcome of *R*_*d*_ on $\theta (\eta )$.

**Figure 10:**
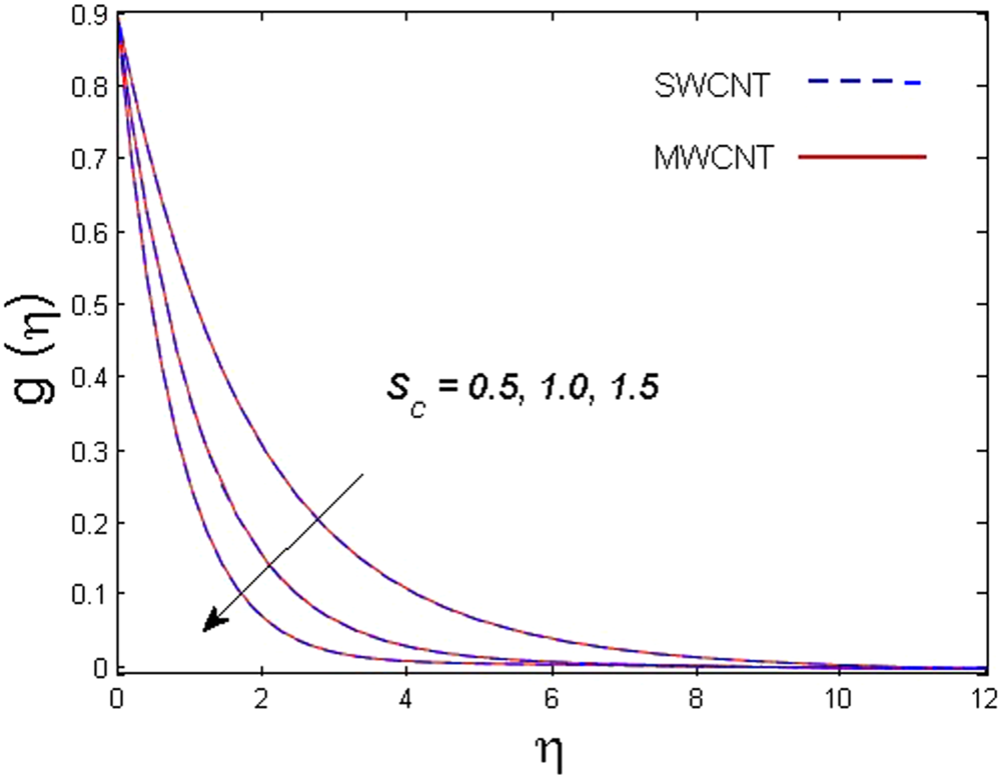
Outcome of *S*_*c*_ on $g(\eta )$.

**Figure 11:**
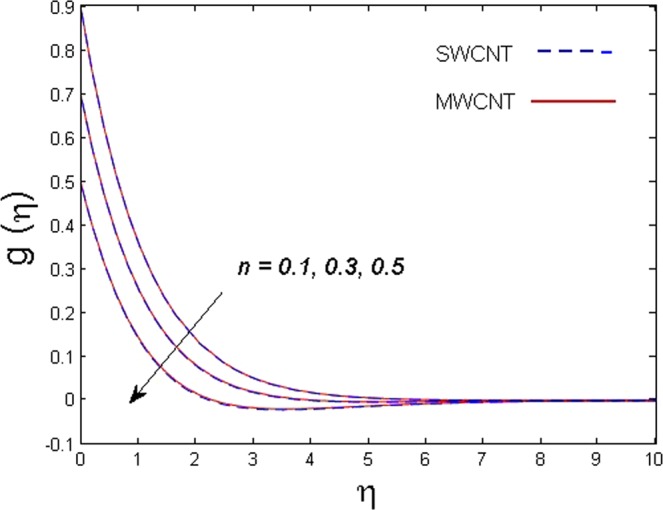
Outcome of *n* on $g(\eta )$.

**Figure 12:**
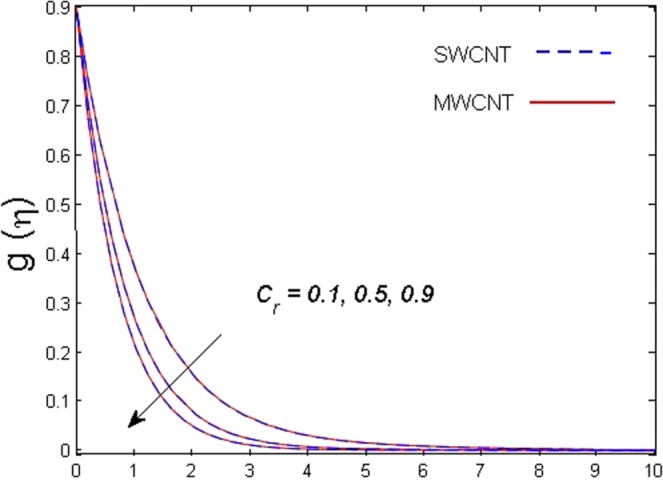
Outcome of *C*_*r*_ on $g(\eta )$.

**Figure 13:**
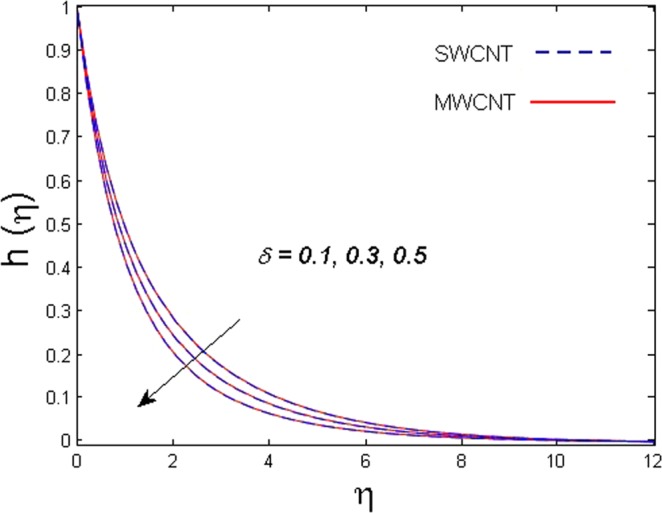
Outcome of *δ* on $h(\eta )$.

**Figure 14:**
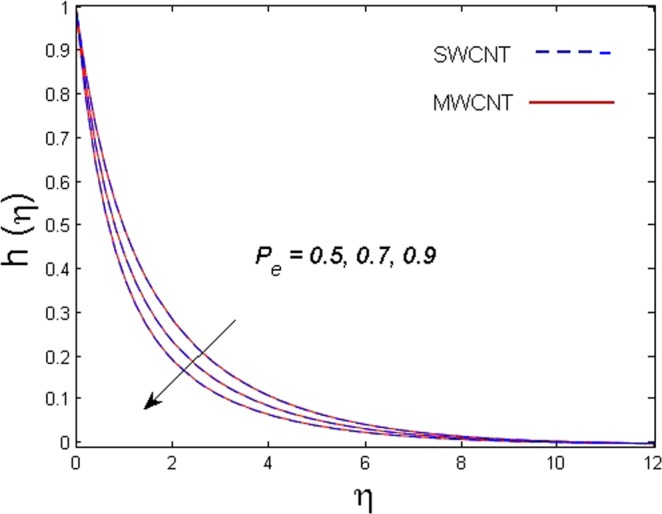
Outcome of *P*_*e*_ on $h(\eta )$.

**Figure 15:**
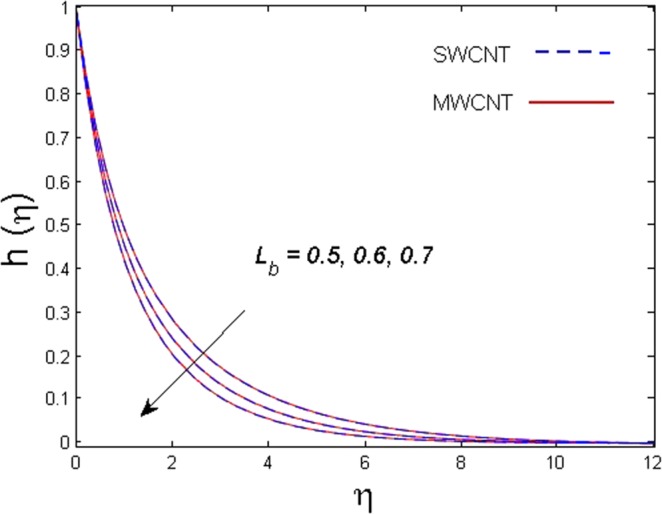
Outcome of *L*_*b*_ on $h(\eta )$.

**Figure 16:**
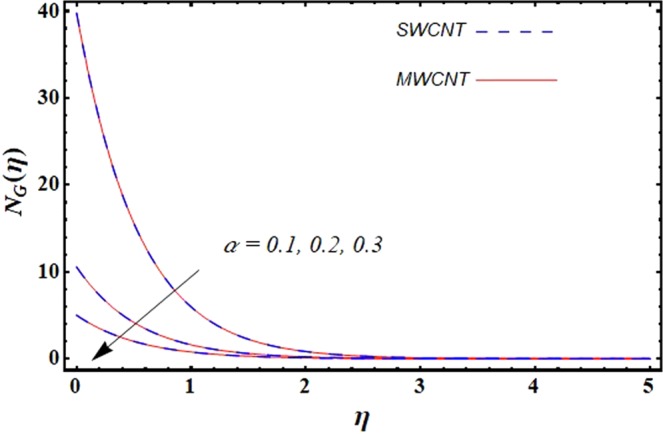
Outcome of *α* on ${N}_{G}(\eta )$.

**Figure 17:**
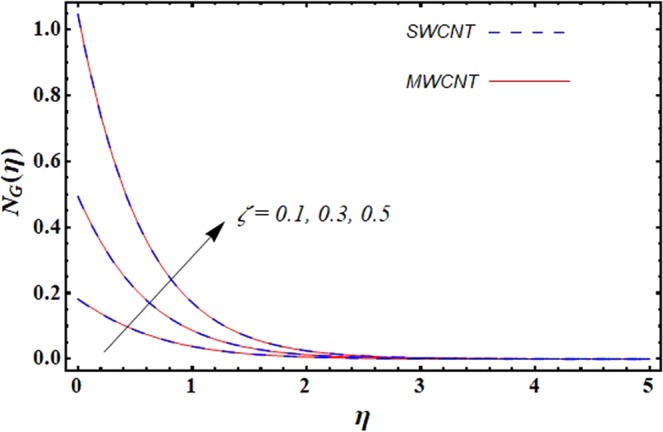
Outcome of *ζ* on ${N}_{G}(\eta )$.

**Figure 18:**
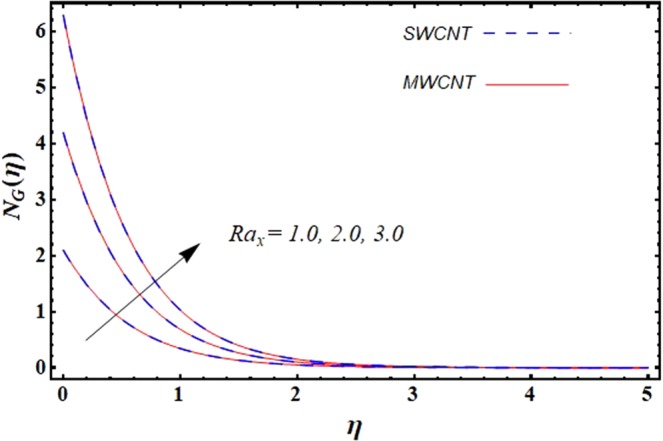
Outcome of *Ra*_*x*_ on ${N}_{G}(\eta )$.

**Figure 19:**
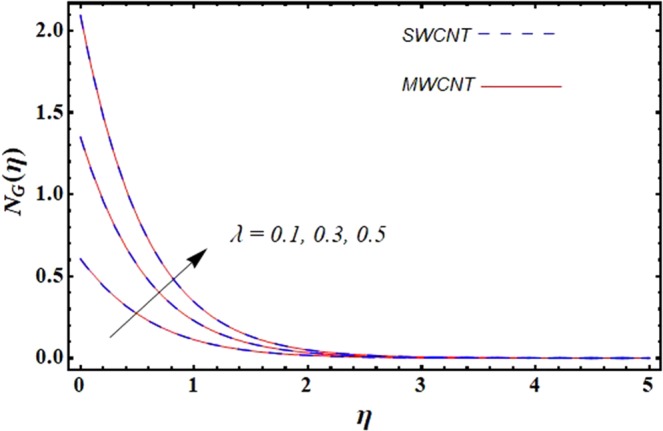
Outcome of *λ* on ${N}_{G}(\eta )$.

This section is dedicated to discuss and anticipate the impacts of various parameters $0.01\le \varphi \le 0.03$, $0.4\le M\le 1.0$, $0.1\le {k}_{1}\le 0.7$, $0.1\le {{\boldsymbol{v}}}_{{\boldsymbol{0}}}\le 0.3$, $0.2\le {N}_{r}\le 1.5$, $0.1\le {R}_{b}\le 0.3$, $0.5\le {B}_{1}\le 1.5$, $0.1\le {R}_{d}\le 0.7$, $0.5\le {S}_{c}\le 1.5$, $0.1\le n\le 0.5$, $0.1\le {C}_{r}\le 0.9$, $0.5\le {P}_{e}\le 0.9$, $0.5\le {L}_{b}\le 0.7$, $0.1\le \delta \le 0.5$, $0.1\le {\alpha }_{c}\le 0.3$, $0.1\le \lambda \le 0.5$, $1.0\le {R}_{x}\le 3.0$, $0.1\le \xi \le 0.5$, on involved distributions. The numerical values of parameters are fixed as given below: $\varphi =0.01$, ${S}_{c}={B}_{1}=1.0=M={V}_{0}$, ${N}_{r}={P}_{e}={k}_{1}=0.5={L}_{b}$, ${R}_{b}=n={R}_{d}=0.1={C}_{r}=\delta$ and $\Pr =6.2$ unless stated separately. Figure 2 demonstrates the action of solid volume fraction $\varphi$ of nanoparticle on axial velocity. The velocity field enhances for augmenting values of the solid volume fraction $\varphi$ for both nanoparticles. It is also understood that the velocity distribution upsurge rapidly for SWCNT in comparison to MWCNT. Figure 3 is portrayed to depict the impact of the magnetic parameter *M* on the velocity field. The velocity of the fluid lessens for boosting values of *M*. It is because the strong Lorentz force that presents resistance to the fluid’s movement that eventually lowers the fluid’s movement. In Fig. 4, the consequence of porous parameter *k*_1_ on velocity profile is sketched. It is comprehended that the velocity is a lessening function of *k*_1_. Further, the momentum boundary layer for both nanoparticles declines while increasing *k*_1_. The influence of suction parameter v_0_ on axial velocity is shown in Fig. 5. It is perceived that the velocity profile diminishes for boosting estimations of the suction parameter v_0_. Also, the momentum boundary layer declines for cumulative values of v_0_ for both SWCNTs and MWCNTs. The buoyancy ratio parameter *N*_*r*_ and bio-convection Rayleigh number *R*_*b*_ effects on axial velocity are examined in Figs 6 and 7. The velocity profile declines with increasing values of *N*_*r*_ and *R*_*b*_. It is also found that the velocity profile for MWCNT decreases more rapidly than SWCNT in both cases. Figure 8 is illustrated to depict the impact of Biot number *B*_1_ on temperature field. An upsurge in temperature of the fluid is visualized for larger estimates of *B*_1_ for both types of CNTs. Higher thermal resistance in comparison to the boundary layer inside the cone is witnessed. As a result, temperature of the fluid in the vicinity of the boundary layer is seen. The thermal radiation parameter *R*_*d*_ effect on the temperature distribution is shown in Fig. 9. More heat is generated with the rise in values of *R*_*d*_. That is why rise in temperature of the fluid is perceived. The outcome of Schmidt number *S*_*c*_ on concentration field is visualized in Fig. 10. The weak concentration of the fluid is seen for higher values of *S*_*c*_. As the Schmidt number is the quotient of kinematic viscosity to molecular diffusion coefficient small values of molecular diffusion coefficient results in large estimates of *S*_*c*_ that lowers the concentration. Figures 11 and 12 are graphed to comprehend the effect of solutal stratification *n* and chemical reaction parameter *C*_*r*_ on the concentration of the fluid. It is witnessed that concentration of the fluid is on decline for higher estimates of *n* and *C*_*r*_ in case of both types of CNTs. Figures 13, 14 and 15 are portrayed to see the impression of bio-convection constant *δ*, bio-convection Peclet number *P*_*e*_ and bio-convection Lewis number *L*_*b*_ on the density of motile microorganism respectively. It is observed that density of motile microorganism is on decline for all three parameters for both types of nanoparticles. Figures 16 to 19 illustrate the impression of different physical parameters versus the entropy generation number *N*_*G*_. From Fig. 16, it is seen that increasing the temperature difference parameter *α*, the entropy generation number *N*_*G*_ decreases for both nanoparticles. Figures 17 and 18 demonstrate the effects of concentration difference *ζ* and Reynold number *Ra*_*x*_ on the entropy generation number. The entropy generation profile enhances with enhancing the value of *Ra*_*x*_ and *ζ* for both nanoparticles. The local entropy generation increases for growing estimates of the diffusive constant parameter *λ* for both SWCNT and MWCNT which is displayed in Fig. 19.

Table 3 is erected for Skin friction coefficient versus numerous estimates of the arising parameters in the defined mathematical model. It is comprehended that Skin friction parameter rises versus suction parameter and solid volume fraction. Likewise, it decreases for the values of the magnetic parameter, bio-convection Rayleigh number, and porous medium. The values of Nusselt number for various values of involved parameters are given in Table 4. It is witnessed that Nusselt number is decreasing function of magnetic parameter and it enhances for radiation parameter, Biot number, and solid volume fraction. Table 5 is erected to witness the behavior of parameters versus the Sherwood number. For the growing estimates of the buoyancy ratio parameter and concentration stratification, Sherwood number is on the decline and grows versus numerical values of the Schmidt number and chemical reaction parameter. To see the impact of certain parameters on Motile density number, Table 6 is formed. It is witnessed that Motile density number escalates for the values of microorganism concentration difference parameter and Peclet number. Whereas it deteriorates for increasing estimates of Rayleigh number.

**Table 3:** Values of Skin friction $\tfrac{1}{{(1-\varphi )}^{2.5}}f^{\prime\prime} (0)$ versus various estimates of different parameters.

*ϕ*	*k*_1_	*V*_0_	*R*_*b*_	*M*	$\tfrac{{\bf{1}}}{{({\bf{1}}-{\boldsymbol{\varphi }})}^{{\bf{2}}.{\bf{5}}}}{\boldsymbol{f}}^{\prime\prime} ({\bf{0}})$	
					SWCNTs	MWCNTs
**0.1**	0.5	1.0	0.1	1.0	0.30353	0.061725
**0.2**					0.32300	0.062952
**0.3**					0.34039	0.066716
	0.2				0.30318	0.062753
	0.3				0.30331	0.063878
	0.4				0.30354	0.065125
		0.5			0.37751	0.043884
		0.6			0.36265	0.023883
		0.7			0.34774	0.003161
			0.2		0.26144	0.258220
			0.3		0.21970	0.216980
			0.4		0.17832	0.176110
				0.5	1.46240	1.254000
				0.6	1.38720	1.193200
				0.7	1.32120	1.138400

**Table 4:** Values of Nusselt number $-\,\frac{{k}_{nf}}{{k}_{f}}(1+{R}_{d})\theta ^{\prime} (0)$ versus various estimates of different parameters.

*ϕ*	*R*_*d*_	*B*_1_	*M*	*Ec*	$-\,\frac{{{\boldsymbol{k}}}_{{\boldsymbol{nf}}}}{{{\boldsymbol{k}}}_{{\boldsymbol{f}}}}({\bf{1}}+{{\boldsymbol{R}}}_{{\boldsymbol{d}}}){\boldsymbol{\theta }}^{\prime} ({\bf{0}})$		
					SWCNTs	MWCNTs	
**0.01**	0.1	1.0	1.0	0.3	0.46994	0.53224	
**0.02**					0.48506	0.55144	
**0.03**					0.50430	0.57400	
	0.2				0.49596	0.56679	
	0.3				0.52110	0.60050	
	0.4				0.54547	0.63345	
		0.5			0.32314	0.35182	
		0.7			0.39319	0.43630	
		1.0			0.46994	0.53224	
			1.0		0.46994	0.53224	
			2.0		0.48446	0.55417	
			3.0		0.49215	0.56583	
				0.1	0.46994	0.46814	
				0.5	0.56501	0.56242	
				1.0	0.60506	0.60231

**Table 5:** Values of Sherwood number −*g*′(0) versus various estimates of different parameters.

*S*_*c*_	*C*_*r*_	*n*	*N*_*r*_	−*g*′(0)	
				SWCNTs	MWCNTs
**0.1**	0.1	0.1	0.5	0.31891	0.31882
**0.5**				0.50221	0.50155
**0.9**				0.74207	0.74087
	0.1			0.80642	0.80511
	0.2			0.88714	0.88613
	0.3			0.95695	0.95612
		0.2		0.73573	0.73379
		0.3		0.66795	0.66532
		0.4		0.60326	0.59988
			0.6	0.79903	0.79771
			0.7	0.79130	0.78997
			0.8	0.78319	0.78185

**Table 6:** Values of Motile density number ${N}{{n}}_{{x}}{R}{{a}}_{{x}}^{-1/4}$ versus various estimates of different parameters.

*L*_*b*_	*P*_*e*_	*R*_*b*_	*δ*	−*h*′(0)	
				SWCNTs	MWCNTs
**0.5**	0.5	0.1	0.1	0.71908	0.71883
**0.6**				0.79036	0.70040
**0.7**				0.86372	0.86334
	0.1			0.45916	0.45897
	0.2			0.52402	0.52381
	0.3			0.58896	0.58874
		0.2		0.75780	0.75713
		0.3		0.75339	0.75276
		0.4		0.74899	0.74840
			0.2	0.74834	0.74807
			0.3	0.77760	0.77732
			0.4	0.80685	0.80657

## Concluding Remarks

The aqueous base nanofluid flow with both types of CNTs (SWCNT and MWCNT) over a vertical cone accompanied by impacts of a gyrotactic microorganism containing motile organisms and solutal stratification in a porous medium is deliberated numerically here. The analysis is performed in the presence of heat generation/absorption, Joule heating, and chemical reaction. An additional effect of Entropy generation is also taken into account. The salient characteristics of the modeled problem are:

- With an increase in estimates of Peclet number, Motile density number enhances.
- For both CNTs, the velocity of the fluid escalates versus growing values of suction and magnetic parameters.
- The fluid’s temperature is on the rise for the estimates of the buoyancy ratio parameter.
- For both types of CNTs, the concentration of the fluid is on the decrease in the values of solutal stratification.
- For the growth estimates of the buoyancy ratio parameter, Sherwood number is on the decline and grows versus numerical values of chemical reaction parameter.
- Skin friction parameter rises for solid volume fraction.
- Nusselt number is decreasing the function of the magnetic parameter.
